# Hierarchical Mergence Approach to Cell Detection in Phase Contrast Microscopy Images

**DOI:** 10.1155/2014/758587

**Published:** 2014-05-28

**Authors:** Lei Chen, Jianhua Zhang, Shengyong Chen, Yao Lin, Chunyan Yao, Jianwei Zhang

**Affiliations:** ^1^College of Computer Science and Technology, Zhejiang University of Technology, Hangzhou 310023, China; ^2^College of Life Sciences, Fujian Normal University, Fuzhou, Fujian 350007, China; ^3^Department of Informatics, University of Hamburg, 22527 Hamburg, Germany

## Abstract

Phase contrast microscope is one of the most universally used instruments to observe long-term cell movements in different solutions. Most of classic segmentation methods consider a homogeneous patch as an object, while the recorded cell images have rich details and a lot of small inhomogeneous patches, as well as some artifacts, which can impede the applications. To tackle these challenges, this paper presents a hierarchical mergence approach (HMA) to extract homogeneous patches out and heuristically add them up. Initially, the maximum region of interest (ROI), in which only cell events exist, is drawn by using gradient information as a mask. Then, different levels of blurring based on kernel or grayscale morphological operations are applied to the whole image to produce reference images. Next, each of unconnected regions in the mask is applied with Otsu method independently according to different reference images. Consequently, the segmentation result is generated by the combination of usable patches in all informative layers. The proposed approach is more than simply a fusion of the basic segmentation methods, but a well-organized strategy that integrates these basic methods. Experiments demonstrate that the proposed method outperforms previous methods within our datasets.

## 1. Introduction

### 1.1. Introduction

In biological research areas, the detection of cells and the identification of life stages are significant. Both of them play critical roles in accessing the effectiveness of anticancer drugs, optimizing the formula of culture solution for cell proliferation and cell pathological analysis, and so forth.

Phase contrast microscope [1, 2] is one of the most common imaging devices, which is used for the acquisition of medical images, especially cell images, without additional fluorescence indicators. For the colorless and transparent cell specimens, phase contrast microscopy system can produce sharp images. Although these images can be manually analyzed, different kinds of cell transformations and morphological states, including proliferation, division, and apoptosis, may increase detecting error rate and make the procedure extremely time-consuming. The judgment criteria may vary in the long span of manual detection, which is unfavorable to the outcome. Therefore, it is necessary to develop a computer-assisted method with a consistent standard to automatically detect individual cells and analyze cell morphologies. All of these make segmentation one of the most challenging tasks for microscopy cell image analyses. Segmentation results also lay the foundation for the further analyses, such as cell tracking and cell stage identification.

**Figure 1:**
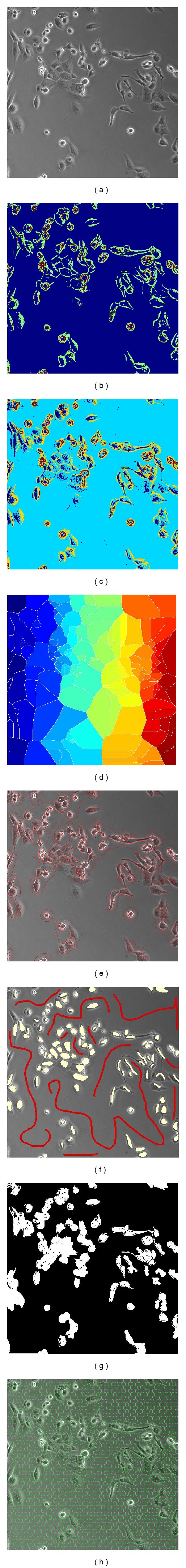
Representation of phase contrast image and some experimental results. (a) The original phase contrast image is labeled as NO.500 in T24_xy6 image sequence of our datasets. The image shown is 1004 × 1004 pixels. (b) Otsu multithresholds (3 classes, 2 thresholds). (c) Otsu multithresholds (4 classes, 3 thresholds). (d) The color labeled result image based on marker-controlled watershed. (e) The outcome of active contour method with an initialization curve on the image boundary. (f) Graph cut initialization. (g) Graph cut result. (h) The result of superpixel method in gray intensity domain.

Due to the phase contrast imaging principle [3], phase contrast microscopic images [1, 4, 5] contain artifacts, that is, bright-halo and shade-off. These two effects make cell images more inhomogeneous. An example is shown in Figure 1(a), where most intracellular areas are at the same grayscale interval as the background. Most of cells in the view are glued together and the boundaries between objects and background are not always evident. Diverse cell morphologies, as well as different ways of cell adhesion, perplex the entire situation. Phase contrast microscope indeed enhances the contrast of intracellular structure, but black and white spots make it hard to get homogenous patches. Moreover, the produced substance along the cell boundary, which is at the same grayscale level as the intracellular areas, can also increase the difficulty of cell segmentation.

### 1.2. Related Work

For many years, a lot of widely used image segmentation methods have been proposed. Thresholds [6, 7] based on grayscale histogram have a long history of application on image segmentation field. However, histogram only contains grayscale statistical information, which cannot represent local structures effectively. Multilevel Otsu method with different number of thresholds is globally applied to segment image into classes. As shown in Figures 1(b) and 1(c), the results are not satisfactory. Edge detection methods [8–10] are well used in some situations where objectives are quite different from the background in grayscale or RGB domain. When the background is similar to the objective, it is hard to locate the real boundary. Watershed method [11, 12] and marker-controlled watershed method [13] take advantage of the local topological structures. Morphological operations [14] are used as auxiliary means in these methods. The result is demonstrated in Figure 1(d), in which the patches cannot be recognized as cell regions. Mean shift [15, 16] is a self-adapting kernel density estimation method. It makes use of local data distribution to estimate a local pattern. Active contour models (ACM) [17–20] evolve curves mainly based on local gray intensity and gradient information. The initialization contour location and the searching direction can influence the outcome to a great extent. When placing the curve on the image boundary with outside-in searching direction, the result can be generated after iterative curve evolution, as shown in Figure 1(e). Some interactive approaches [21, 22] mainly based on graph cut method [23, 24] and superpixel method [25] are more suitable for the natural colored image segmentation. Referring to Figures 1(f) and 1(g), the interactive graph cut method needs a lot of manual labeling work and the result image contains many linked regions that cannot represent individual cells.  Figure 1(h) shows the result of superpixel method applied in the gray intensity domain. There are about 2500 superpixels in this image. Some of them can bind cell regions, while most cannot. Although it can merge minor patches to get bigger region, most of superpixels are the compound of background and cell parts, or even edges. Recently, Yin et al. [1, 26] proposed a novel approach to segment cell images by treating phase contrast image as a special case. They model the phase contrast microscopy system and formulate it as global quadratic optimization problem. They also applied the superpixel method [25] in the phase feature domain [27] to gain phase-homogeneous atoms.

Since different methods show their advantages and weaknesses in different datasets or under different application contexts, we give a small discussion on some commonly used medical image segmentation methods. They have something in common, which should be given enough attention and can help to address the challenges.

Firstly, for methods based on thresholds [6, 7], the target is to find one or more proper thresholds. Specific thresholds will define subareas and patches of the whole image, and the gray-intensity of each subarea will be restricted in a minor bias from each grayscale center.

Secondly, the ACM [17–20, 28] has been modified and has many versions for specific applications. The ACM aims at evolving the curve until it reaches the stop criteria. It makes use of not only the intensity information but also the local image gradient. In the original version, each pixel will be treated equally, and no bias is allowed. One of the versions allows a small bias given by Gaussian kernel. Then all bounded areas within the small bias will be treated as objects.

Thirdly, the method based on imaging model [1, 26, 27], which formulates optimization functions on the basis of real imaging system. The goal of the method is to minimize the quadratic optimization object function, which combines the spatial term, temporal term, penalty term, and the basic term that is the distance between real images and the target images. The key of the method is the phase of the wave, which can be manipulated and counted as feature. Each phase will be represented by a kernel, which can be interpreted as a point spreading function (PSF). Each kernel gives a bias same as Gaussian kernel. After reaching convergence, each area will be treated as object.

In summary, the general character between all methods presented here is the given bias. The methods treat each area in a small bias as an object and later resort to machine learning to obtain desired patches. And the applied kernel is also significant. Kernel can be interpreted as how the pixel affects its surrounding pixels or how the surrounding pixels affect the central pixel.

### 1.3. Our Proposal

Based on the analyses of some classic methods, this paper presents a simple but efficient method to segment cells in phase contrast microscopy, which is applicable for adhesion situations. Most of steps are based on the classic methods, but we apply them in a different way. During the extraction step, hierarchical strategies are applied to build raw segmentation results. Our novel method can detect all kinds of cells at different stages; then it uses machine learning methods to eliminate noncell blobs. In the procedure, morphological operations are applied universally. However, our method is semiautomatic. Some steps need manual presetting, but most parameters used in the processing flow are relatively fixed.

The contribution of this paper is threefold. The first one is the design of a novel segmentation framework. Hierarchical mergence approach (HMA) takes advantage of hierarchical informative layers to get satisfactory segmentation results.

The second one is that we evaluate different mergence strategies under the HMA framework to get different combination, and the best can be picked as the result.

The third one is the extensive evaluation of the state-of-the-art methods in our datasets, which are more challenging than the experimental datasets in the literature, where our method achieves a significant improvement.

The remainder of this paper is organized as follows.  Section 2 presents the proposed HMA.  Section 3 provides extensive experimental results and the comparison between HMA and previous methods within our datasets.  Section 4 concludes the paper and gives a discussion of future work.

## 2. Methods

**Figure 2:**
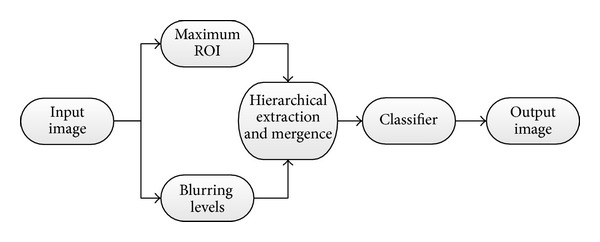
Illustration of HMA framework.

The proposed HMA method can be interpreted by the framework shown in Figure 2. In this framework, the maximum region of interest (ROI) block gives the maximum mask to cover all cell regions, while the blurring levels block gives different reference images based on blurring levels and grayscale morphological operations. The hierarchical extraction and mergence block is the most important part in this framework. At last, wrong patches will be filtered by classifier block.

### 2.1. Maximum ROI

This step only takes the gradient information into consideration to obtain the maximum region of interest. According to our dataset, the intracellular gray-intensity is quiet similar to the solution background, which means cell areas cannot be distinguished by grayscale range.

However, intracellular areas are full of minor structures and inconsistent minor patches, which have a lot of edge information, and the background is comparatively smoother than cell regions. Therefore, the gradient information is sufficient to distinguish cell regions and background regions.

There are many edge detection methods, including Canny [9] and Roberts. For example, in [8], Robert edge operator is applied to medical images to extract regions along with morphological dilation. In practice, most of edge detectors can be utilized, so far as to get the maximum ROI after binary morphological operations [14].

In this paper, Canny operator is applied to the original image *I*(*x*, *y*) and two downsampled images, that is, *I* ^1/2^(*x*, *y*) and *I* ^1/4^(*x*, *y*).

The Canny operator can be formulated as

(1) $$\begin{array}{c} M\left(x,y\right)=\sqrt{G_{x}{\left(x,y\right)}^{2}+G_{y}{\left(x,y\right)}^{2}},\\ \theta \left(x,y\right)=\arctan\left(\frac{G_{x}\left(x,y\right)}{G_{y}\left(x,y\right)}\right), \end{array}$$

where *M*(*x*, *y*) is edge strength matrix, *θ*(*x*, *y*) is edge direction matrix, (*x*, *y*) is the pixel coordinate value in the image spatial domain, and

(2) Gx=[f(x+1,y)−f(x,y)+f(x+1,y+1)−f(x,y+1)]2,Gy=[f(x,y+1)−f(x,y)+f(x+1,y+1)−f(x+1,y)]2.

After applying Canny operator to different scale images, all gradient maps are merged to the original image. The merged map *G*_map_ can be defined as

(3) $$\begin{array}{c} G_{\text{map}}=M_{L}^{1}+M_{L}^{1/2}+M_{L}^{1/4}, \end{array}$$

where the superscript numbers, {1,1/2,1/4}, indicate the downsampled scale and the subscript *L* means the output binary map.

**Figure 3:**
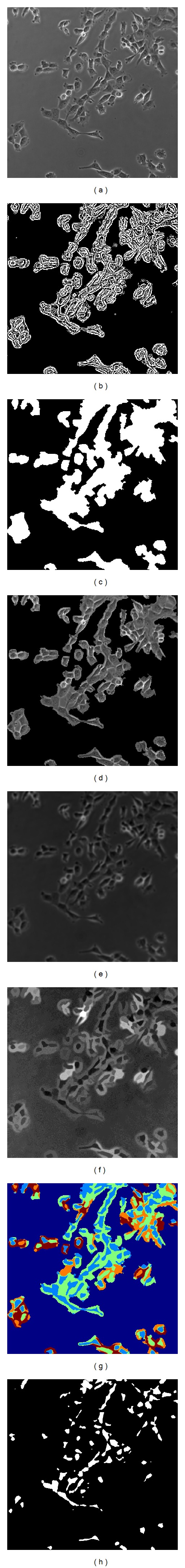
(a) The original phase contrast image is labeled as NO.100 in T24_xy1 image sequence of our datasets. (b) The merged binary map *G*_map_. (c) The maximum binary ROI mask *M*_ROI_. (d) The truncated image based on maximum ROI mask. (e) Blurred image based on Gaussian kernel. (f) Blurred image based on Figure 3(e) with gray morphological operations. (g) After applying Otsu method with three thresholds locally referring to Figure 3(f). (h) After applying watershed method to the blue section of Figure 3(g).

There are many morphologically processing flows that can generate the maximum ROI binary mask. In our model, morphological close, erosion, and area open operations are used, as well as some logical operations. The output maximum ROI binary mask, as shown in Figure 3(c), can cover all cell events as shown in Figure 3(d). Although there are still many regions of background in the mask, this will not influence the application of further steps.

### 2.2. Blurring

Referring to the analyses of previous methods, blurring is really necessary. With the application of blurring [29], the minor structures and inconsistent patches can be fused together, and the local pattern can be more obvious.

Two categories of methods are widely used for blurring images. The first one is the most used methods, which are based on predefined kernels, like Gaussian blurring, mean blurring, Laplacian blurring, and so forth. The second category is diffraction pattern kernel (DPK) [26, 27], which is derived from the phase contrast imaging system.

In this study, Gaussian blurring is chosen due to its simplicity and effectiveness. We apply it with a radius of 5 pixels and a variance of 1.0. It can weaken edges and build consistent patches.

The Gaussian blurring can be reformulated as

(4) $$\begin{array}{c} S^{n}\left(x,y,\sigma \right)={\left[G\left(x,y,\sigma \right)\right]}^{n}*I\left(x,y\right), \end{array}$$

where *G*(*x*, *y*, *σ*) = (1/(2*πσ* ^2^))*e* ^−(*x*^2^ + *y*^2^)/2*σ*^2^^ and the superscript *n* indicates the times of convolution.

**Figure 4:**
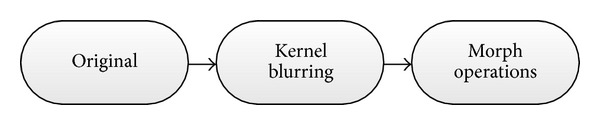
Reference images in the blurring levels section of HMA framework.

Blurring operation makes the visible intracellular structure fuzzy, blends the intracellular prone-dark parts together, and also helps to mix up the prone-bright cell boundaries. Besides kernel-based blurring, there is another way to build consistent areas, which is the grayscale morphological operations [14]. The method is well applied in the marker-controlled watershed. It makes full use of morphological operations to blend the scene. The image, Figure 3(e), is the blurred result when the number *n* is 20, while the image, Figure 3(f), is based on Figure 3(e) with gray morphological operations. Most of consistent patches can be seen clearly in Figure 3(f). The reference images can be generated by the flow in Figure 4.

### 2.3. Local Otsu

We cannot apply Otsu method [6, 7] to the whole image range because of different images along the whole sequence contain different ratio of gray-intensity levels. Otherwise, there will be different segmented results for different images, and the targets cannot be recognized from these segments.

In the maximum ROI mask drawn from the first step, there are many 8-connected individual patches, which can be indicated as *M*_ROI_ = {*M*_ROI_^1^, *M*_ROI_^2^,…, *M*_ROI_^*m*^} and *m* is the total number of individual regions. Each of patches is simply applied with Otsu method locally in this step.

Based on the first two steps, all patches are considered to be only having cell events. So for each patch, the ratio of intensity levels varies in a small scale, and Otsu method with three thresholds can easily separate *M*_ROI_^*i*^, where *i* = 1,…, *m*, into the four classes, that is, dark-section, prone-dark-section, bright-section, and prone-bright-section. Thus, *M*_ROI_ also can be indicated by *M*_ROI_ = {*A*_1_, *A*_2_, *A*_3_, *A*_4_}, the four sections. As shown in Figure 3(g), the dark-section is marked by blue, the prone-dark-section is marked by green, the bright-section is marked by orange, and the prone-bright-section is marked by crimson, while the dark-blue-section represents the background.

### 2.4. Watershed

Watershed method is normally used to separate adherent cells in our study. In [8], the cells are adhered in one direction in its experimental images; that is, for each cell its surrounding cells have no adhesion situations. Thus, watershed method can be applied to its dataset directly to separate adhesion patches into individuals among the global region.

For images in our dataset, cells also adhere in one direction after using Otsu method locally. Thus the watershed method is also applicable to the linked regions. Sometimes, watershed method may produce oversegmentation and undersegmentation cases but in most situations can correctly separate adherent cells based on the local morphological structures. In our model, watershed method is applied universally in the whole processing flow.

Figure 3(h) is the watershed result of the dark-section (or blue-section) in Figure 3(g), which can almost represent and cover cells in the original image. However, some cells are ignored in Figure 3(h), which are wrapped in other colored layers in Figure 3(g). More minor and homogenous patches are to be extracted in the next step.

### 2.5. Patch Extraction and Mergence

**Figure 5:**
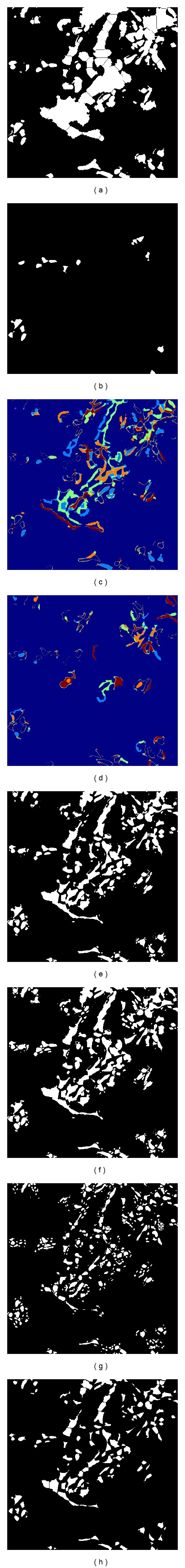
More intermediate results. The original phase contrast image is shown in Figure 3(a). (a) After applying watershed method on the combination image of the blue and green regions in Figure 3(g). (b) The combination of the holes in the orange and crimson regions of Figure 3(g). (c) After applying Otsu method with three thresholds locally to the green section of Figure 3(g). (d) After applying Otsu method with three thresholds locally to the orange section of Figure 3(g). (e)–(g) All three binary images called raw segmentation results are generated by three different strategies under HMA framework. (h) The classification result of Figure 5(e) after SVM filtering.

In this step, a hierarchical approach is proposed to extract more informative patches and merge them into a raw segmentation result. There are many intermediate results as shown in Figure 5.  Figure 5(a) is obtained by applying watershed method on the combination image of the green and blue regions in Figure 3(g). Different patches in the intermediate images have different representations. Some patches in Figure 3(h) can represent main parts of cells, while some patches in Figure 5(a) can cover more areas of individual cells. In Figure 3(g), the orange and crimson sections separate cell patches. Some hollow areas in it can represent cell regions as shown in Figure 5(b). Both Figures 5(c) and 5(d) are colored images after applying Otsu method locally. The aim of producing the colored images in Figure 5 is to get minor areas. These areas that can represent individual cells may be neglected in the preceding procedures.

Before building the raw result, let us make more detailed explanations by referring to Figures 3 and 5. In the presented HMA framework, *M*_ROI_ = {*A*_1_, *A*_2_, *A*_3_, *A*_4_}. After the implementation of multilevel Otsu method with three thresholds locally, *A*_1_ also can be separated into four parts, which can be indicated by *A*_1_ = {*A*_1_ *A*_1_, *A*_1_ *A*_2_, *A*_1_ *A*_3_, *A*_1_ *A*_4_}. Thus, *A*_2_ *A*_1_ represents the blue section in Figure 5(c). Other ways of combination are also useful, such as *A*_2_ *A*_1_ ∪ *A*_2_ *A*_2_, which merges two parts into one ROI, and (*A*_2_ *A*_3_∪ *A*_2_ *A*_4_)_holes_, which indicates the hole areas.

**Figure 6:**
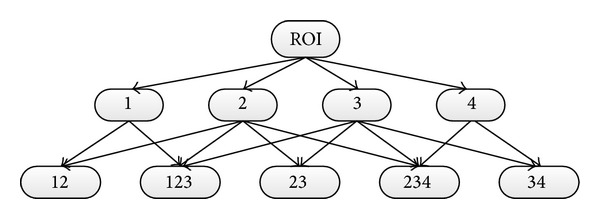
Combination strategy diagram.

Following this way, the combination strategy diagram can be shown as in Figure 6. Different combination strategies try to ensure that the cell target can be masked by a reasonable patch. However, combination strategies of three parts are seldom implemented in our model.

In Figure 6, all nodes in the combination strategy diagram can replace the ROI node to get more informative layers, and the reference images can be changed to get minor patches. The numbers, 1 to 4, represent the four classes after applying Otsu method locally and the combination of these classes is represented by a sequence of numbers. When applying Otsu method each time, the number of thresholds can be manipulated, empirically three. More reference images can be added and used during the procedure but mostly based on Figures 3(e) and 3(f).

At different steps of the procedure, the application domain of multilevel Otsu method is changed, so minor patches can be segmented out. The raw result is produced by the combination of all informative and useful layers, but the strategy to different images may vary.

The raw result, Figure 5(e), is combined mainly by *A*_1_, (*A*_3_ *A*_3_∪ *A*_3_ *A*_4_)_holes_, *A*_3_ *A*_1_, (*A*_4_ *A*_3_∪ *A*_4_ *A*_4_)_holes_, *A*_4_ *A*_1_ and *A*_4_ *A*_2_. However, Figure 5(g) digs deeper and patches in the view are smaller. During the combination steps, some logical operations and biological operations are applied to address the overlapping problems or to expand the hollow areas.

According to our experimental tests, there will be no usable patches after digging three times or more. However, it depends, for the image full of cells, after applying blurring step, on areas more likely to be blended together in large range. In order to obtain the details in the large area, one more application is necessary.

Since different images have different cell distributions and different ratios of cells in different stages, different mergence strategies are applied to different images. From Figure 5(e) to Figure 5(g), three binary images generated by different strategies are demonstrated under HMA framework. Among the three images, there are many different patches, which can complement each other to get a better region covering. After getting the raw segmentation results, Figure 5(e) is picked as the preferred one. Before sending it into SVM classifier [30], watershed method is applied to split the regions. The output image after SVM filtering is shown in Figure 5(h). However, there are still many redundant patches and errors in the image. Discussion on automatic classification problem will be given in Section 3.

## 3. Experiments and Discussion

**Figure 7:**
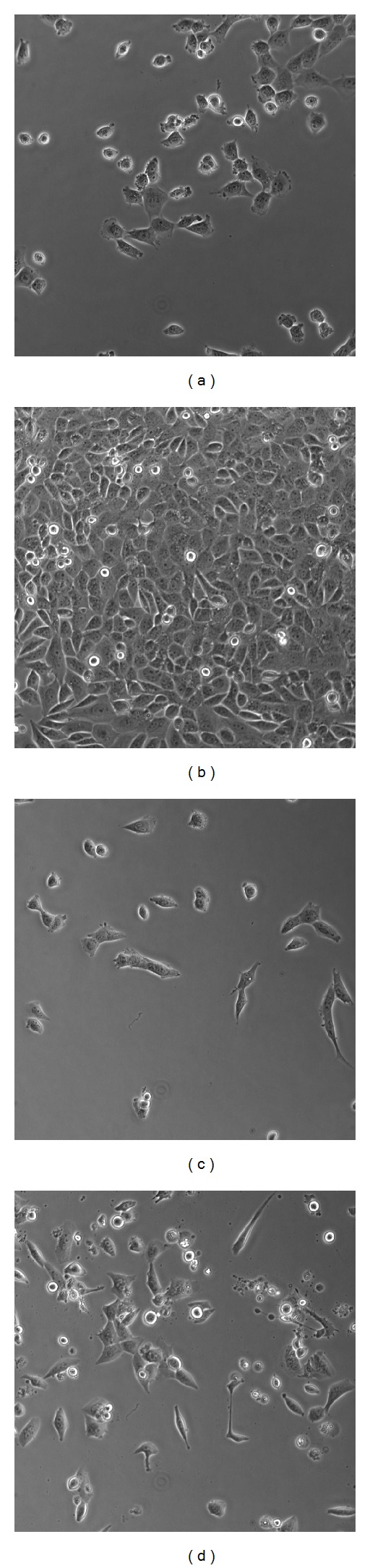
Four more phase contrast images. (a) and (b) labeled as NO.1 and NO.871 in T24_xy1 image sequence, respectively. (c) and (d) labeled as NO.1 and NO.871 in T24_xy6 image sequence, respectively.

Five more sample images from T24_xy6 and T24_xy1 image sequences of our datasets are taken into experiments. In Figure 7, from left to right, the images are labeled as xy1_1, xy1_871, xy6_1, and xy6_871, respectively. The fifth image is in Figure 9(a), which is labeled as xy1_266 in our dataset. Sequence T24_xy1 records the procedure of cell proliferation, while sequence T24_xy6 records the procedure of cell transformation after adding drugs, in which we have apoptosis events. Almost all cells in the view are different in the morphological feature or life stage.

In this part, firstly more classic methods are applied to the sample images and then our experimental results are demonstrated based on the proposed HMA. Next, SVM classifier is applied. At last, some discussions are given.

### 3.1. Experimentation Based on Some Previous Methods

**Figure 8:**
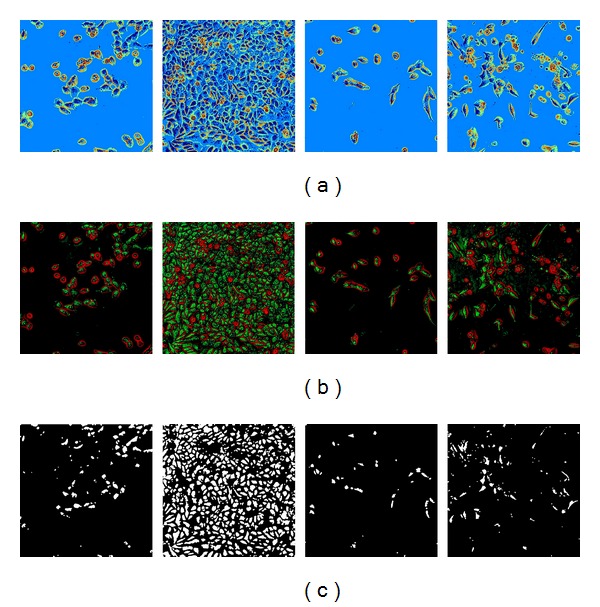
Cell segmentation and detection by some previous methods, PART1. Row 1: after applying Otsu method globally. Row 2: after applying restoration method based on imaging model. Row 3: segmentation results based on the green layer of Row 3 with morphological open operation.

Referring to Figure 8, in Row 1, globally applying Otsu method can separate individual cells into scattered parts but cannot produce patches to represent cells independently. Otsu method utilized relative value to select thresholds, which is the ratio between the number of pixels at a specific level and the total number of pixels in the image. Otsu method may produce unexpected segmentation results if the number of thresholds is wrongly assigned.

In Row 2, after applying restoration method based on imaging model [1, 26] only, the green parts almost can represent normal cells, but mitosis and apoptosis cells cannot be masked by specific patches. Since Otsu method equally treats all pixels, the restoration method incorporates DPK, which gives a small tolerance. So, to some extent, the green parts can match with the dark-blue parts in Row 1.

The binary images in Row 3 are the results after applying morphological dilation operations based on the green patches in Row 2. The images are counted as raw segmentation results in [1].

**Figure 9:**
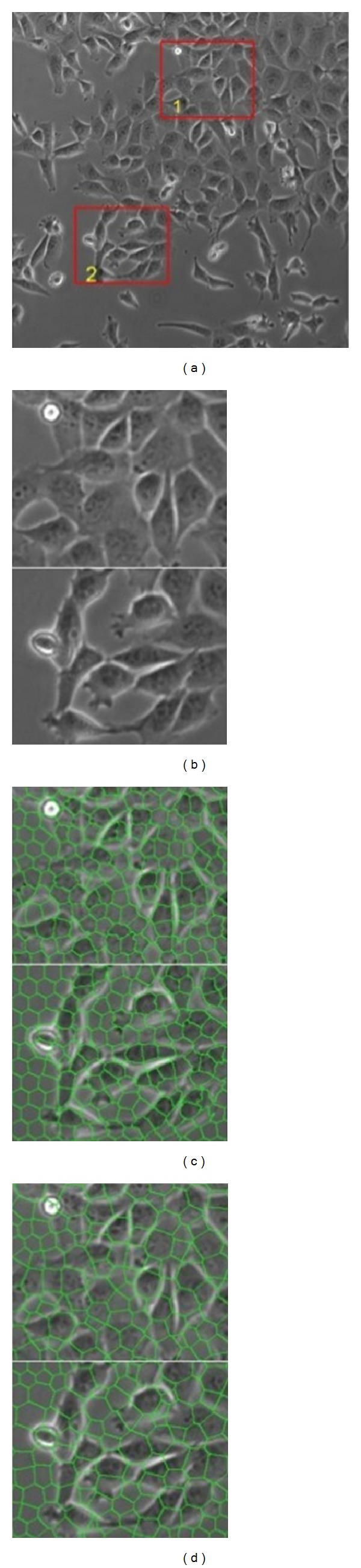
Cell segmentation and detection by some previous methods, PART2. (a) The original phase contrast image, labeled as NO.266 in T24_xy1 image sequence. (b) The selected two regions (both regions are bounded by red rectangles with yellow marked numbers). (c) Local atoms based on intensity. (d) Local atoms based on phase features.

In Figure 9, experimental results of SLIC superpixel framework [25] on different feature domains are demonstrated. In this paper, the criterion of building phase homogeneous atoms is different from that of [27]. The total number of superpixels in the original image is about 2500.

In the intensity domain, each patch has its own grayscale consistency, but it is still hard to merge nearby patches to consist a whole region to represent an individual cell. Some of patches cover both the edge and the intracellular parts, while some are difficult to distinguish.

In the phase feature domain, the patches became larger. However, there is also no obvious clue to merge patches and they cannot be easily classified into predefined classes. The linked patches that actually belong to the different cells can also have the same consistency. Although the superpixel method can get smaller patches when presetting number is changed, the local structure can be damaged.

### 3.2. Experimentation Based on the Proposed HMA

This paper conducts experiments with different combination strategies under HMA framework and some alternative processing flows are determined. The processing flows use different informative layers and patches. For each image, different processing flows can produce different results. The optimal segmentation result can be manually picked.

**Figure 10:**
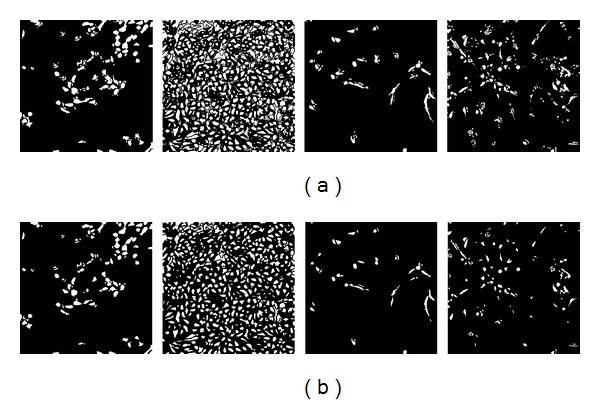
Results based on HMA framework. Row 1: four raw segmentation results (four original images are shown in Figure 7). Row 2: after SVM classification based on Row 1.

Comparing to images in Row 3 of Figure 8, images in Figure 10 are the combination of more informative lower layers and patches than the restoration method based on imaging model [1]. The restoration based on imaging model method can gain information only from the output RGB three-layer image, which will make it difficult to get minor patches to represent minor objects. Cell phase contrast images contain many cell objects. When applying with interactive methods, there are many markers to be signed, which may increase more label works per image.

Among the experimental results based on HMA, redundant patches are drawn, which are noncell patches. In order to delete them, this paper resorts to morphological features, local binary pattern (LBP) [31] feature, and also phase features [27] to screen the cell target patches out. Compared with the results after classification and the raw results, most of noncell regions are deleted, but they still have errors. For T24_xy1 sequence, the average cell level segmentation accuracy is 76.3%. For T24_xy6 sequence, the average cell level segmentation accuracy is 73.2%.

### 3.3. Discussion

Judging all figures in this paper, the output segmentation results cannot strictly cover individual cells; only the main parts of cells can be covered by masks. According to the results of the proposed HMA method, all patches are smaller than the ground truth. There are many factors that result in such outcomes; faint boundary in original images and the selected kernel are the most significant ones. And the imprecise segmented patches can lead to the failure of classification. These all contribute to the low segmentation accuracy.

Considering the traits of our datasets, it is hard to enhance the boundary; a better way of getting homogeneous patches is to pick a more descriptive kernel. The kernel can merge cell parts together to a consistent gray-level. In this paper, we simply pick the Gaussian kernel to merge different parts.

The restoration based on imaging model gives us a new perspective, but when it comes to cell images with intracellular inhomogeneous areas, the small valued DPK cannot help to get all patches. Compared with the dataset in [1, 27] and the dataset in this paper, the images in our dataset are more inconsistent and more challenging, while the images in the previous papers are either homogeneity prone or cell region separated. Generally, the proposed method can get relatively good segmentation results on our datasets.

## 4. Conclusion

Since not all situations are satisfied with the prerequisites of all methods, we screen out the applicable ones and organize them in an ingenious way. What we do in different steps of HMA is to fit to the preconditions of each classic method, taking advantage of all methods at large. Thus, the key point of this paper is that applying methods after satisfying all prerequisites strictly. The goal of the later drawn mergence step is to get the smaller cell regions and more descriptive regions, but they cannot exceed the cell maximum boundary.

The presented hierarchical mergence approach could perform under relatively fixed extracting flows to obtain the final results. On further study, stronger inference strategies and more automatic methods will be incorporated in the framework. The proposed method has been applied to our ten raw image sequences. The experimental results can fit the requirement to some extent, but redundant patches may influence the later tracking procedure. Our group will enhance the hierarchical mergence framework.
